# A streamlined model for use in clinical breast cancer risk assessment maintains predictive power and is further improved with inclusion of a polygenic risk score

**DOI:** 10.1371/journal.pone.0245375

**Published:** 2021-01-22

**Authors:** Richard Allman, Erika Spaeth, John Lai, Susan J. Gross, John L. Hopper

**Affiliations:** 1 Genetic Technologies / Phenogen Sciences, Fitzroy, Australia; 2 Phenogen Sciences, Charlotte, North Carolina, United States of America; 3 Centre for Epidemiology and Biostatistics, The University of Melbourne, Parkville, VIC, Australia; University of Chicago, UNITED STATES

## Abstract

Five-year absolute breast cancer risk prediction models are required to comply with national guidelines regarding risk reduction regimens. Models including the Gail model are under-utilized in the general population for various reasons, including difficulty in accurately completing some clinical fields. The purpose of this study was to determine if a streamlined risk model could be designed without substantial loss in performance. Only the clinical risk factors that were easily answered by women will be retained and combined with an objective validated polygenic risk score (PRS) to ultimately improve overall compliance with professional recommendations. We first undertook a review of a series of 2,339 Caucasian, African American and Hispanic women from the USA who underwent clinical testing. We first used deidentified test request forms to identify the clinical risk factors that were best answered by women in a clinical setting and then compared the 5-year risks for the full model and the streamlined model in this clinical series. We used OPERA analysis on previously published case-control data from 11,924 Gail model samples to determine clinical risk factors to include in a streamlined model: first degree family history and age that could then be combined with the PRS. Next, to ensure that the addition of PRS to the streamlined model was indeed beneficial, we compared risk stratification using the *Streamlined model* with and without PRS for the existing case-control datasets comprising 1,313 cases and 10,611 controls of African-American (n = 7421), Caucasian (n = 1155) and Hispanic (n = 3348) women, using the area under the curve to determine model performance. The improvement in risk discrimination from adding the PRS risk score to the *Streamlined model* was 52%, 46% and 62% for African-American, Caucasian and Hispanic women, respectively, based on changes in log OPERA. There was no statistically significant difference in mean risk scores between the *Gail model plus risk PRS* compared to the *Streamlined model plus PRS*. This study demonstrates that validated PRS can be used to streamline a clinical test for primary care practice without diminishing test performance. Importantly, by eliminating risk factors that women find hard to recall or that require obtaining medical records, this model may facilitate increased clinical adoption of 5-year risk breast cancer risk prediction test in keeping with national standards and guidelines for breast cancer risk reduction.

## Introduction

Apart from non-melanoma skin cancer, breast cancer is the most common form of cancer affecting women and approximately one in eight women in the United States of America (U.S.A) will develop the disease in their lifetime [1]. In 2019, an estimated 268,000 U.S. women were diagnosed with invasive breast cancer and approximately 41,000 will have died as a result. There is, therefore, a need to identify which women are more likely to develop sporadic disease, so as to best apply measures to prevent it.

For women who are not initially identified as at high risk based on previous personal history of breast cancer, or family history suggestive of germline pathogenic mutations, the U.S. Preventative Services Task Force (USPSTF) [2], the American Society of Clinical Oncology (ASCO) [3], as well as the National Comprehensive Cancer Network (NCCN) [4], all have guidelines that these women should be screened to determine their five-year risk of developing breast cancer and offered risk-reducing medications, if appropriate.

This is a responsibility that falls upon the woman’s primary care health professional, as these patients would not fall under the initial ‘high risk’ rubric. The USPSTF uses a 5-year high-risk threshold of 3% and recommends a strong grade B (‘offer or provide this service’) guidance that providers offer tamoxifen or raloxifene for women above this 3% threshold [2], while ASCO and NCCN use a lower 5-year high-risk threshold of 1.67%, and in addition to tamoxifen and raloxifene, provide the option of aromatase inhibitors [3, 4].

Approximately 10 million women in the U.S.A are eligible for breast cancer risk reducing medication [5]. Even though uptake of risk reducing medications has been reportedly low [6, 7], the majority of eligible women are not being assessed for their risk. Multiple tools are available to provide the risk assessment, including the Gail Model [8, 9] and the Breast Cancer Surveillance Consortium Risk Calculator [10], but they tend to be underutilized for various reasons [11] including lack of routine risk assessment and time constraints. Furthermore, there is often a failure to complete risk factor questionnaires, especially for the Gail model [12]. The overall result being that many women (and their physicians) may be unaware of their risk of developing breast cancer and that preventive options are available. These options are not just risk reducing medications, but also include increased surveillance and lifestyle modifications, such as reduction of alcohol consumption, increasing exercise, and maintaining a healthy body weight.

Indeed, whilst 96% of physicians agree that assessing breast cancer risk was a primary care provider’s responsibility, 76% never calculate a Gail score [13]. Surprisingly, over 70% of internal medicine residents reported no knowledge of the Gail model [14]. In real-world clinical practice, a “streamlined model” relying on only the age and family history of a woman is more likely being utilized subconsciously by the physician in absence of a full model. Use of the Gail model can also be problematic because of its reliance on women remembering clinical information that may have occurred many years prior. It is also of note that the question of whether the woman has had at least one breast biopsy with atypical hyperplasia requires a level of understanding of medical terminology that most patients would not have. Of note, it is known that women with atypical hyperplasia are at increased risk of breast cancer based on their biopsy status alone and their risk score is actually underestimated by the Gail model [15].

With the advent of genome wide association studies (GWAS), researchers have identified single nucleotide polymorphisms (SNP) that are risk markers for breast cancer that are independent of clinical risk factors [16, 17]. In the same paper that validated the SNP set used for risk assessment, the authors found that just using two easily accessible clinical risk factors, that of family history and age, combined with this SNP set provided a superior risk assessment model [18]. However, this paper focused on a 10-year score, while the US guidelines are built around a 5-year risk score assessment. Based on the above, a compelling case could be made for developing a more streamlined 5-year risk model that would help providers be compliant with national standards, using a similar model of limited but proven risk factors and risk SNP.

Therefore, the purpose of this study was twofold: (1) To develop a streamlined 5-year risk assessment tool based on validated risk SNP (PRS) that incorporates clinical risk factors from the Gail model that are readily available and important to risk prediction and (2) to evaluate the strength of this test’s 5-year risk prediction capabilities.

## Materials and methods

### Clinical review sample

De-identified test request forms from 2,339 African American, Caucasian, and Hispanic U.S.A women who had been tested with the BREVAGen*plus* (Phenogen Sciences) commercial breast cancer risk assessment test, between October 2014 and October 2016, were reviewed to determine how many of the Gail model questions were not answered or answered as “unknown”. This analysis was reviewed by an independent institutional review board and deemed exempt (Quorum Review IRB). For model comparison, we removed the patient samples with unknown family history results (n = 57) for final sample size of 2882. Sample characteristics and summary can be found in S1 and S2 Tables, respectively.

### Model validation sample

An independent dataset consisting of a total of 1,313 case and 10,611 controls from two different cohorts were used to compare the performance of the two models. Details of the 7,421African American (416 case/7005 control), 1,155 Caucasian (750 case/405 control), and 3,348 Hispanic (147 case/3210 control) women used in the risk discrimination analyses are described elsewhere [19, 20]. Briefly, the Caucasian women were identified from the Australian site of the Breast Cancer Family Registry and the African American and Hispanic women were identified from the Women’s Health Initiative (WHI) SNP Health Association Resource (SHARe). Women with unknown family history were removed from the analysis. Our SNP were validated in African American and Hispanic women [19], however population-specific SNP improvements need be made in future studies for a more robust model because the majority of the SNP panels were discovered in European ancestry populations.

The Gail model, or the NCI’s breast cancer risk assessment tool (BCRAT), is a well-established risk prediction tool that incorporates age, age at menarche, age at parity, 1^st^ degree family history, and biopsy status including presence of atypical hyperplasia. This clinical gold-standard was the model upon which the initial commercial clinical test was built: Gail+PRS [19, 20].

### Polygenic risk score and combined risk score

Using the approach of Mealiffe et al. [21], we calculated a PRS—a SNP-based (relative) risk score using previously published estimates of the odds ratio (OR) per allele and risk allele frequency (*p*) assuming independent and additive risks on the log OR scale (S3–S5 Tables). For each SNP, we calculated the unscaled population average risk as μ = (1 –*p*)^2^ + 2*p* (1 –*p*) OR + p^2^OR^2^. Adjusted risk values (with a population average risk equal to 1) were calculated as 1/μ, OR/μ and OR^2^/μ for the three genotypes defined by number of risk alleles (0, 1, or 2). The overall SNP-based risk score was then calculated by multiplying the adjusted risk values for each of the 70+ SNP.

We created a five-year absolute clinical risk of breast cancer based on published relative risks for having an affected first-degree relative [22], and taking into account the competing risk of dying from other causes. Ethnic-specific breast cancer incidence and competing mortality data were derived from the U.S.A SEER database (SEER 2013 Research Data). Absolute 5-year risk is calculated using the following formula:

$$abs\text{\_}risk\text{\_5}=\frac{\text{(}cumul\text{\_}b\text{\_5}-cumul\text{\_}b\text{)}\times mortsurv\text{\_5}}{\text{(1-}cumul\text{\_}b\text{)}}$$

Where cumul_b is the cumulative risk at baseline (*cumul*_*b* = 1 – *e*^−*fh* × *snp* × *incid_b*^)

And cumul_b_5 is the cumulative risk at baseline plus 5-years (*cumul*_*b*_5 = 1 – *e*^−*fh* × *snp* × *incid*_*b*_5^)

Where;

incid_b is the cumulative incidence of breast cancer from birth to baseline,

incid_b_5 is the cumulative incidence of breast cancer from birth to baseline plus 5 years,

mortsurv_5 is survival from baseline age to baseline age plus 5 years

fh is family history relative risk

snp is the SNP-based relative risk score calculated using the method of Mealiffe [21].

In developing a streamlined Gail model, we elected to retain only a patient’s age and first-degree family history of breast cancer, which do not require recall of events over long periods of time.

Combined absolute five-year risk scores based on Gail model plus PRS or the *Streamlined model plus PRS*, were calculated as previously described [19, 20].

### Statistical analysis

For the series of test request forms, we used descriptive statistics to assess the completeness of the data (Table 1). Comparative analyses of five-year risk estimate between the *Gail Model plus PRS* versus the *Streamlined model plus PRS* were performed using the log transformation of the aforementioned commercial clinical samples (n = 2,882).

**Table 1:** Missing data from Gail model questions in 2,339 U.S. women who have undergone commercial breast cancer risk testing.

Gail model questions	% of women with missing information, or “unknown” answers	% of women with a positive response
Patient Age	0.0%	100%
Age at menarche	4.4%	95.7%
Age at time of first live birth	1.3%	78.5%
family history*	2.4%	40.3%
Ever had a breast biopsy	1.1%	34.1%
How many breast biopsies	2.4%	34.1%
Atypical hyperplasia^+^	4.0%	4.9%
Ethnicity	0.0%	100%

* Any first-degree relative with breast cancer

^+^ At least one biopsy with atypical hyperplasia

For the case-control data, we used logistic regression to estimate the change in log odds per adjusted standard deviation (OPERA) for log-transformed age-adjusted five-year risks [23]. We used the log OPERA and the area under the receiver operator curve (AUC) to assess risk discrimination. All tests were two sided and p-values <0.05 were considered nominally statistically significant. Stata Release 14 [24] was used for all statistical analyses.

## Results and discussion

### Clinical commercial sample study discovery

The extent to which Gail model questions were not answered or answered as “unknown” was assessed to determine how often data went missing from the test inputs. Our data set of the aforementioned 2,339 women indicates that approximately 16% of all answers relating to the Gail model were not answered, or answered as “unknown”, as part of their risk testing. The most commonly unanswered question was age of menarche, with 4.4% of women being unable to provide an answer (Table 1). The second most common unanswered question (or answered “unknown”) related to whether the patient had at least one biopsy with atypical hyperplasia (Table 1). There was no missing information for age and ethnicity.

Based on the above, we developed a *Streamlined Model*, requiring only the patient’s age and first-degree family history of breast cancer, with the assumption that these do not require long term recall (for example, age of first menses), nor access to medical records (number of biopsies and/or diagnosis of atypical hyperplasia).

**Fig 1:**
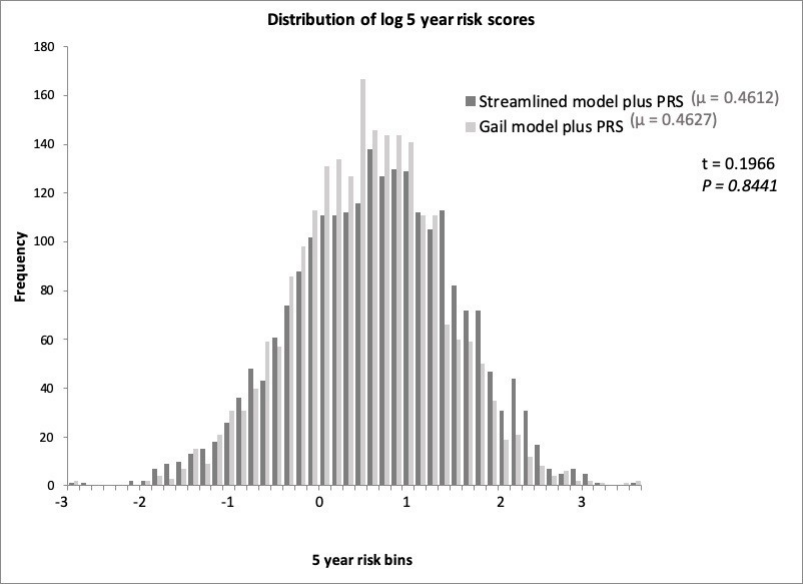
Distribution of patient samples when risk scores calculated with Gail model plus PRS compared to the Streamlined models plus PRS. Log-transformed values of the five-year risk distributions and the t-test results for the risk estimates obtained by the *Gail model plus PRS* versus the *Streamlined model plus PRS*. The t-test indicates that there is no difference in mean values between the *Streamlined model plus PRS* and *Gail model plus PRS* (p = 0.8441).

A comparative analysis of five-year risk estimates between the *Gail model plus PRS* versus the *Streamlined model plus PRS*, was performed using the 2,339 commercial samples (excluding patients for whom the 1st degree relative response was missing or unknown, n = 57) (Fig 1). The two-tailed t-test of the log transformed absolute 5-year risk scores between the *Gail model plus PRS* compared to the *Streamlined model plus PRS* indicates that there is no significant (n = 2282; P = 0.8441) difference in mean risk scores between each model (Fig 1).

### Case-control study validation

Using a combined multi-ethnic case-control dataset, we assessed the extent to which adding a PRS to the *Streamlined model* improved breast cancer risk prediction compared with predictions using the *Streamlined mo*del alone. Table 2 indicates that AUCs were highest for risk prediction when using the *Streamlined model* in conjunction with PRS, with values of 0.57 (95% CI = 0.54, 0.60) for African Americans, 0.64 (95% CI = 0.61, 0.67) for Caucasians, and 0.60 (95% CI = 0.55, 0.65) for Hispanics. Similarly, the OPERA across all ethnicities for the combined risk scores (*Streamlined model plus PRS* = age, family history, ethnicity and PRS) were higher than both the PRS alone, and the *Streamlined model* risk score alone (Table 2). Importantly, we previously published the AUC for the AA and His case/control using the Gail and the Gail plus SNP [19]. While we recognize that the *Streamlined model plus PRS* has a modest decrease in AUC compared to the published Gail plus SNP model, the Gail model, or any model with multiple clinical risk factors is being underutilized in clinic. The modest decrease in AUC may be an acceptable outcome if clinicians are able to risk assess a greater percentage of their patient population. Although there are African American and Hispanic specific SNP in their respective PRS within this model [19], there is still significant room for improvement. Shieh et al. [25] published a pooled case control analysis of Latina women wherein PRS improvement was seen. Likewise, GWAS studies in women of African ancestry have further identified SNP that may increase PRS AUC [26, 27]. The next step is to integrate these markers into a risk model and cross validate in an independent cohort. As the collaborative consortium continue to expand, the opportunity to more accurately include other and mixed genetic ancestries will be imperative.

**Table 2:** AUC and OPERA values, with 95% confidence intervals (CI), for different risk prediction models in different race/ethnicity.

	AUC	(95% CI)	OPERA	95% CI	p
African American (n = 7421)					
PRS only	0.555	(0.525, 0.584)	1.241	(1.123, 1.371)	<0.001
1st-degree family history (*y/n)	0.531	(0.499, 0.562)	1.203	(1.100, 1.317)	<0.001
1st-degree family history and PRS risk score	0.570	(0.539, 0.601)	1.316	(1.190, 1.455)	<0.001
Caucasian, non-Hispanic (n = 1155)					
PRS only	0.612	(0.597,0.646)	1.458	(1.292, 1.645)	<0.001
1st-degree family history (*y/n)	0.586	(0.552,0.620)	1.387	(1.213, 1.585)	<0.001
1st-degree family history and PRS risk score	0.639	(0.606, 0.672)	1.614	(1.416, 1.838)	<0.001
Hispanic (n = 3348)					
PRS only	0.590	(0.543, 0.636)	1.390	(1.177, 1.643)	<0.001
1st-degree family history (*y/n)	0.547	(0.499, 0.594)	1.226	(1.058, 1.420)	0.007
1st-degree family history and PRS risk score	0.601	(0.554, 0.647)	1.484	(1.255, 1.756)	<0.001

* y/n = yes/no.

The increments in AUCs are similar to the increments in log OPERAs, and showed that the improvements in risk prediction from including the PRS were approximately 7.5%, 8.5% and 9.1% for African Americans, Caucasians, and Hispanics, respectively for the AUCs and 52%, 46% and 62% for the log OPERAs.

## Conclusions

Breast cancer PRS is an underutilized risk factor that can add value to the clinical implementation of risk assessment in the general population. In an effort to improve the clinical application of breast cancer risk scores in the general population, we have streamlined a clinical risk model (Gail) by retaining just age and whether a first-degree relative has breast cancer, and then including PRS, consistent with the approach taken by Mavaddat et al. [18] These clinical variables are important risk factors for breast cancer as cancer increases substantially with age [1] and the presence of a first degree relative with breast cancer is associated with, an approximate doubling of a woman’s risk [22]. Given the lack of full breast cancer risk assessment at the primary care level [13], physicians are presumably relying on only age and first degree family history of breast cancer as a subconscious measure of risk for their patient—similar to this *Streamlined model*. Importantly, we focus on the 5-year risk score and not the lifetime risk score with this *Streamlined model* because it is well established in clinical recommendations that the Gail model does not include enough family history to appropriately determine lifetime risk for screening guidance [4, 28, 29]. Furthermore, the majority of women diagnosed with sporadic breast cancer typically have little or no family history. Therefore, most women in the general population will have a lifetime risk score that will never surpass the 20% threshold of actionable risk based on PRS and age alone [30].

We have incorporated polygenic risk of over 70 SNP to the *Streamlined model plus PRS* to provide an absolute five-year breast cancer risk prediction to improve performance beyond these simple clinical risk factors alone. In terms of differentiating women who will develop breast cancer from those who will not develop breast cancer, adding a PRS to the *Streamlined model* is on average 53% better than the *Streamlined model* alone across the three ethnicities in this study. Our OPERA and ROC analysis indicates an average 8.4% increase in AUC and 50% increase in log OPERA values with the addition of PRS to the *Streamlined model*. Clearly, the more information that is incorporated into a risk model, the more accurate that model will be. However, model accuracy is dependent on the accuracy of the input. Of interest, our data suggests that questions such as age at menarche and age at first live birth culminate in a low relative contribution to the overall risk score. When looking at 5-year risk scores, the predictive ability of two important, common clinical risk factors plus PRS (*Streamlined model plus PRS*) is similar for the *Gail model plus PRS*, with a reduction in the mean AUC of only 0.02. Thus, our data indicate that reducing the Gail questionnaire to only two clinical variables maintains the integrity of a breast cancer risk prediction algorithm in a clinical setting when PRS is included. This streamlined questionnaire could make it easier for physicians to administer an absolute five-year risk assessment for the majority of women who do not meet other high-risk criteria.

Our *Streamlined model plus PRS* is designed for assessing 5-year breast cancer risk in women who are not yet categorized as “high risk.” Because the Gail model has been shown to previously underestimate risk in women with atypia [15, 31], we did not include that clinical factor into our *Streamlined model plus PRS*. Furthermore, we acknowledge that NCCN guidelines suggest women with atypical hyperplasia are categorized as high risk based on biopsy confirmed atypia alone [4]. Interestingly, we observed a statistically significant difference (p<0.005) between PRS from commercial patients with atypical hyperplasia (n = 112) versus patients with no biopsy history (n = 1503; S1 and S2 Tables). This suggests a possible modest association between PRS and atypical hyperplasia that could be further exploited to improve risk assessment prior to the point of biopsy.

As PRS continue to improve, so will the breast cancer risk assessment models. There exists solid evidence on additional SNP that could further improve the *Streamlined model plus PRS* [30, 32]. Unfortunately, due to the clinical restrictions on our commercial samples, we did not have the ability to retrospectively assess alternative SNP to the 77 initially included in our interrogation.

By increasing breast cancer risk assessment of the general population, physicians can increase patient breast cancer awareness and identify those patients at increased risk of breast cancer enabling a more proactive breast health management, potentially improving compliance with current guidance on risk reduction [2].

## Supporting information

S1 Table These data represent the 2282 commercial clinical samples that were used in the initial study discovery. These samples were collected between October 2014 and October 2016 and were genotyped based on ethnicity (77 for Cau; 75 for AA; 71 for His). The original commercial model incorporated the Gail model +PRS (for which 5 year and lifetime risk score can be found in column O and P; calculated per Dite, 2016). The Gail model risk scores alone can be found in columns M and N. The Streamlined model plus PRS risk scores are in columns Q and R. The polygenic risk scores are in column L were calculated per Mealiffe 2010. The clinical Gail model questions can be found in columns D-K, respectively: current age; age at menarche, age at 1st live birth; previous biopsy; number of previous biopsies; confirmed atypical hyperplasia; ethnicity.
(XLSX)

S2 Table Characteristics for commercial samples (n = 2282). 57 samples with unknown family histories were removed from the initial 2339 samples for the model comparison because family history is a major component of both models.
(DOCX)

S3 Table Unadjusted ORs for individual SNPs in Caucasians. (DOCX)

S4 Table Unadjusted ORs for individual SNPs in African Americans. (DOCX)

S5 Table Unadjusted ORs for individual SNPs in Hispanics. (DOCX)

## References

[1] Siegel R.L., Miller K.D., and Jemal A. (2019). Cancer statistics, 2019. CA Cancer. J. Clin. 69, 7–34. 10.3322/caac.21551 30620402

[2] US Preventive Services Task Force, Owens D.K., Davidson K.W., Krist A.H., Barry M.J., Cabana M., et al (2019). Medication Use to Reduce Risk of Breast Cancer: US Preventive Services Task Force Recommendation Statement. Jama 322, 857–867. 10.1001/jama.2019.11885 31479144

[3] Visvanathan K., Hurley P., Bantug E., Brown P., Col N.F., Cuzick J., et al (2013). Use of pharmacologic interventions for breast cancer risk reduction: American Society of Clinical Oncology clinical practice guideline. J. Clin. Oncol. 31, 2942–2962. 10.1200/JCO.2013.49.3122 23835710

[4] NCCN. National Comprehensive Cancer Network. NCCN Cinical Practice Guidelines in Oncology. Breast Cancer Risk Reduction V1.2019. Last Updated, December 2018; Available at: https://www.nccn.org/professionals/physician_gls/pdf/breast_risk_blocks.pdf. Accessed, April, 6 2019.

[5] Freedman A.N., Graubard B.I., Rao S.R., McCaskill-Stevens W., Ballard-Barbash R., and Gail M.H. (2003). Estimates of the number of US women who could benefit from tamoxifen for breast cancer chemoprevention. J. Natl. Cancer Inst. 95, 526–532. 10.1093/jnci/95.7.526 12671020

[6] Ropka M.E., Keim J., and Philbrick J.T. (2010). Patient decisions about breast cancer chemoprevention: a systematic review and meta-analysis. J. Clin. Oncol. 28, 3090–3095. 10.1200/JCO.2009.27.8077 20458026 PMC2903338

[7] Smith S.G., Sestak I., Forster A., Partridge A., Side L., Wolf M.S., et al (2016). Factors affecting uptake and adherence to breast cancer chemoprevention: a systematic review and meta-analysis. Ann. Oncol. 27, 575–590. 10.1093/annonc/mdv590 26646754 PMC4803450

[8] Gail M.H., Brinton L.A., Byar D.P., Corle D.K., Green S.B., Schairer C., et al (1989). Projecting individualized probabilities of developing breast cancer for white females who are being examined annually. J. Natl. Cancer Inst. 81, 1879–1886. 10.1093/jnci/81.24.1879 2593165

[9] Costantino J.P., Gail M.H., Pee D., Anderson S., Redmond C.K., Benichou J., et al (1999). Validation studies for models projecting the risk of invasive and total breast cancer incidence. J. Natl. Cancer Inst. 91, 1541–1548. 10.1093/jnci/91.18.1541 10491430

[10] Tice J.A., Cummings S.R., Smith-Bindman R., Ichikawa L., Barlow W.E., and Kerlikowske K. (2008). Using clinical factors and mammographic breast density to estimate breast cancer risk: development and validation of a new predictive model. Ann. Intern. Med. 148, 337–347. 10.7326/0003-4819-148-5-200803040-00004 18316752 PMC2674327

[11] Crew K.D. (2015). Addressing barriers to uptake of breast cancer chemoprevention for patients and providers. Am. Soc. Clin. Oncol. Educ. Book e50-8. 10.14694/EdBook_AM.2015.35.e50 25993215 PMC7276203

[12] Sherman M.E., Ichikawa L., Pfeiffer R.M., Miglioretti D.L., Kerlikowske K., Tice J., et al (2016). Relationship of Predicted Risk of Developing Invasive Breast Cancer, as Assessed with Three Models, and Breast Cancer Mortality among Breast Cancer Patients. PLoS One 11, e0160966 10.1371/journal.pone.0160966 27560501 PMC4999085

[13] Sabatino S.A., McCarthy E.P., Phillips R.S., and Burns R.B. (2007). Breast cancer risk assessment and management in primary care: provider attitudes, practices, and barriers. Cancer Detect. Prev. 31, 375–383. 10.1016/j.cdp.2007.08.003 18037249

[14] Yadav S., Hartkop S., Cardenas P.Y., Ladkany R., Halalau A., Shoichet S., et al (2019). Utilization of a breast cancer risk assessment tool by internal medicine residents in a primary care clinic: impact of an educational program. BMC Cancer 19, 228-019-5418-6. 10.1186/s12885-019-5418-6 PMC6416938 30871497

[15] Pankratz V.S., Hartmann L.C., Degnim A.C., Vierkant R.A., Ghosh K., Vachon C.M., et al (2008). Assessment of the accuracy of the Gail model in women with atypical hyperplasia. J. Clin. Oncol. 26, 5374–5379. 10.1200/JCO.2007.14.8833 18854574 PMC2651072

[16] Maas P., Barrdahl M., Joshi A.D., Auer P.L., Gaudet M.M., Milne R.L., et al (2016). Breast Cancer Risk From Modifiable and Nonmodifiable Risk Factors Among White Women in the United States. JAMA Oncol. 2, 1295–1302. 10.1001/jamaoncol.2016.1025 27228256 PMC5719876

[17] Milne R.L., Gaudet M.M., Spurdle A.B., Fasching P.A., Couch F.J., Benitez J., et al (2010). Assessing interactions between the associations of common genetic susceptibility variants, reproductive history and body mass index with breast cancer risk in the breast cancer association consortium: a combined case-control study. Breast Cancer Res. 12, R110 10.1186/bcr2797 21194473 PMC3046455

[18] Mavaddat N., Pharoah P.D., Michailidou K., Tyrer J., Brook M.N., Bolla M.K., et al (2015). Prediction of breast cancer risk based on profiling with common genetic variants. J. Natl. Cancer Inst. 107, 10.1093/jnci/djv036 Print 2015 May. 25855707 PMC4754625

[19] Allman R., Dite G.S., Hopper J.L., Gordon O., Starlard-Davenport A., Chlebowski R., et al (2015). SNPs and breast cancer risk prediction for African American and Hispanic women. Breast Cancer Res. Treat. 154, 583–589. 10.1007/s10549-015-3641-7 26589314 PMC4661211

[20] Dite G.S., MacInnis R.J., Bickerstaffe A., Dowty J.G., Allman R., Apicella C., et al (2016). Breast Cancer Risk Prediction Using Clinical Models and 77 Independent Risk-Associated SNPs for Women Aged Under 50 Years: Australian Breast Cancer Family Registry. Cancer Epidemiol. Biomarkers Prev. 25, 359–365. 10.1158/1055-9965.EPI-15-0838 26677205 PMC4767544

[21] Mealiffe M.E., Stokowski R.P., Rhees B.K., Prentice R.L., Pettinger M., and Hinds D.A. (2010). Assessment of clinical validity of a breast cancer risk model combining genetic and clinical information. J. Natl. Cancer Inst. 102, 1618–1627. 10.1093/jnci/djq388 20956782 PMC2970578

[22] Collaborative Group on Hormonal Factors in Breast Cancer. (2001). Familial breast cancer: collaborative reanalysis of individual data from 52 epidemiological studies including 58,209 women with breast cancer and 101,986 women without the disease. Lancet 358, 1389–1399. 10.1016/S0140-6736(01)06524-2 11705483

[23] Hopper J.L. (2015). Odds per adjusted standard deviation: comparing strengths of associations for risk factors measured on different scales and across diseases and populations. Am. J. Epidemiol. 182, 863–867. 10.1093/aje/kwv193 26520360 PMC4757943

[24] StataCorp. (2016). Stata statistical software, release 14. Statacorp Lp.

[25] Shieh Y., Fejerman L., Lott P.C., Marker K., Sawyer S.D., Hu D., et al (2020). A Polygenic Risk Score for Breast Cancer in US Latinas and Latin American Women. J. Natl. Cancer Inst. 112, 590–598. 10.1093/jnci/djz174 31553449 PMC7301155

[26] Huo D., Feng Y., Haddad S., Zheng Y., Yao S., Han Y.J., et al (2016). Genome-wide association studies in women of African ancestry identified 3q26.21 as a novel susceptibility locus for oestrogen receptor negative breast cancer. Hum. Mol. Genet. 25, 4835–4846. 10.1093/hmg/ddw305 28171663 PMC5975608

[27] Ruiz-Narváez E.A., Sucheston-Campbell L., Bensen J.T., Yao S., Haddad S., Haiman C.A., et al (2016). Admixture Mapping of African-American Women in the AMBER Consortium Identifies New Loci for Breast Cancer and Estrogen-Receptor Subtypes. Front. Genet. 7, 170 10.3389/fgene.2016.00170 27708667 PMC5030764

[28] Ozanne E.M., Drohan B., Bosinoff P., Semine A., Jellinek M., Cronin C., et al (2013). Which risk model to use? Clinical implications of the ACS MRI screening guidelines. Cancer Epidemiol. Biomarkers Prev. 22, 146–149. 10.1158/1055-9965.EPI-12-0570 23093547

[29] NCCN. National Comprehensive Cancer Network. NCCN Cinical Practice Guidelines in Oncology. Breast Cancer Screening and Diagnosis V3.2018. Last Updated, October 2018; Available at: https://www.nccn.org/professionals/physician_gls/pdf/breast-screening.pdf. Accessed April, 6 2019.

[30] Mavaddat N., Michailidou K., Dennis J., Lush M., Fachal L., Lee A., et al (2019). Polygenic Risk Scores for Prediction of Breast Cancer and Breast Cancer Subtypes. Am. J. Hum. Genet. 104, 21–34. 10.1016/j.ajhg.2018.11.002 30554720 PMC6323553

[31] Hartmann L.C., Degnim A.C., Santen R.J., Dupont W.D., and Ghosh K. (2015). Atypical hyperplasia of the breast—risk assessment and management options. N. Engl. J. Med. 372, 78–89. 10.1056/NEJMsr1407164 25551530 PMC4347900

[32] Michailidou K., Lindstrom S., Dennis J., Beesley J., Hui S., Kar S., et al (2017). Association analysis identifies 65 new breast cancer risk loci. Nature 551, 92–94. 10.1038/nature24284 29059683 PMC5798588

