# Supplementary material for: A streamlined model for use in clinical breast cancer risk assessment maintains predictive power and is further improved with inclusion of a polygenic risk score
Source: PLoS One. 2021 Jan 22;16(1):e0245375. doi: 10.1371/journal.pone.0245375 (PMC7822550; doi:10.1371/journal.pone.0245375)
Supplement: S2 Table — 57 samples with unknown family histories were removed from the initial 2339 samples for the model comparison because family history is a major component of both models. (DOCX) [file pone.0245375.s002.docx]

S2 Table. Characteristics for commercial samples (n=2282). 57 samples with unknown family histories were removed from the initial 2339 samples for the model comparison because family history is a major component of both models.

| Population characteristics (n=2282)^ |  |
| --- | --- |
| Average age | 49.9 (s= 8.76) |
| % with 1^st^ degree FH | 41.28% |
| % w/Live birth | 78.60% |
| Average Age at 1^st^ birth | 27.7 (s=6.75) |
| Average Age at menarche | 12.6 (s=1.63) |
| % w/Any biopsy | 34.18% |
| Average # biopsies | 1.67 (s=1.22) |
| % of all women with Atypical hyperplasia, [% of women with biopsy-history with AH] | 4.91% [14.4%] |
| % Ethnicity (% AA/Cau/His) | AA 6.3% |
|  | Cau 85.4% |
|  | His 8.3% |
| Average PRS: all (n=2282) | 1.19 (s=0.572) |
| Average PRS (no/unk biopsy history, n=1502) | 1.17 (s=0.566) |
| Average PRS (yes biopsy history, n=780) | 1.22 (s=0.584) * |
| Average PRS (AH+ biopsy, n=112) | 1.33 (s=0.698)** |
| ^2282 = original 2339 patient population prior to 57 women removed with unknown 1st degree FH status |  |
| *p<0.05 between average PRS for women with biopsies compared to no biopsies | |
| **P<0.005 between average PRS for women with AH compared to no biopsies | |
