# Supplementary material for: A streamlined model for use in clinical breast cancer risk assessment maintains predictive power and is further improved with inclusion of a polygenic risk score
Source: PLoS One. 2021 Jan 22;16(1):e0245375. doi: 10.1371/journal.pone.0245375 (PMC7822550; doi:10.1371/journal.pone.0245375)
Supplement: S3 Table — (DOCX) [file pone.0245375.s003.docx]

S3 Table. Unadjusted ORs for individual SNPs in Caucasians

| **SNP** | **Chromosome** | **Alleles^*^** | **OR^†^ (95% CI) *P*** | **Published OR^a^** |
| --- | --- | --- | --- | --- |
| rs616488 | 1 | A/G | 0.79 (0.66, 0.95) 0.01 | 1.06 |
| rs11552449 | 1 | C/T | 1.24 (0.99, 1.56) 0.06 | 1.08 |
| rs11249433 | 1 | A/G | 0.99 (0.83, 1.17) 0.9 | 1.10 |
| rs6678914 | 1 | G/A | 0.99 (0.83, 1.17) 0.9 | 1.01 |
| rs4245739 | 1 | A/C | 1.29 (1.05, 1.58) 0.02 | 1.03 |
| rs12710696 | 2 | G/A | 0.98 (0.83, 1.16) 0.8 | 1.04 |
| rs4849887 | 2 | C/T | 0.83 (0.63, 1.09) 0.2 | 1.09 |
| rs2016394 | 2 | G/A | 0.93 (0.79, 1.10) 0.4 | 1.05 |
| rs1550623 | 2 | A/G | 0.85 (0.67, 1.08) 0.2 | 1.06 |
| rs1045485 | 2 | G/C | 0.88 (0.69, 1.13) 0.3 | 1.04 |
| rs13387042 | 2 | A/G | 0.78 (0.66, 0.93) 0.005 | 1.14 |
| rs16857609 | 2 | C/T | 1.00 (0.82, 1.21) 1.0 | 1.07 |
| rs6762644 | 3 | A/G | 1.12 (0.94, 1.34) 0.2 | 1.07 |
| rs4973768 | 3 | C/T | 0.97 (0.82, 1.15) 0.7 | 1.09 |
| rs12493607 | 3 | G/C | 0.98 (0.82, 1.18) 0.9 | 1.05 |
| rs9790517 | 4 | C/T | 1.03 (0.83, 1.28) 0.8 | 1.05 |
| rs6828523 | 4 | C/A | 0.86 (0.65, 1.13) 0.3 | 1.10 |
| rs10069690 | 5 | C/T | 0.93 (0.77, 1.12) 0.4 | 1.02 |
| rs7726159 | 5 | C/A | 1.05 (0.87, 1.26) 0.6 | 1.04 |
| rs2736108 | 5 | C/T | 0.96 (0.80, 1.16) 0.7 | 1.07 |
| rs10941679 | 5 | A/G | 0.94 (0.77, 1.14) 0.5 | 1.12 |
| rs889312 | 5 | A/C | 1.23 (1.02, 1.49) 0.03 | 1.12 |
| rs10472076 | 5 | T/C | 1.04 (0.87, 1.25) 0.6 | 1.04 |
| rs1353747 | 5 | T/G | 0.87 (0.65, 1.16) 0.4 | 1.09 |
| rs1432679 | 5 | A/G | 1.16 (0.97, 1.37) 0.1 | 1.07 |
| rs11242675 | 6 | T/C | 0.88 (0.73, 1.05) 0.1 | 1.06 |
| rs204247 | 6 | A/G | 1.01 (0.85, 1.20) 0.9 | 1.05 |
| rs17529111 | 6 | A/G | 0.99 (0.80, 1.21) 0.9 | 1.05 |
| rs12662670 | 6 | T/G | 1.01 (0.75, 1.38) 0.9 | 1.14 |
| rs2046210 | 6 | G/A | 1.17 (0.98, 1.41) 0.09 | 1.05 |
| rs720475 | 7 | G/A | 0.91 (0.74, 1.10) 0.3 | 1.06 |
| rs9693444 | 8 | C/A | 1.11 (0.93, 1.34) 0.2 | 1.07 |
| rs6472903 | 8 | T/G | 0.94 (0.74, 1.18) 0.6 | 1.10 |
| rs2943559 | 8 | A/G | 1.07 (0.78, 1.48) 0.7 | 1.13 |
| rs13281615 | 8 | A/G | 1.31 (1.10, 1.57) 0.003 | 1.10 |
| rs11780156 | 8 | C/T | 1.27 (1.02, 1.57) 0.03 | 1.07 |
| rs1011970 | 9 | G/T | 1.15 (0.91, 1.45) 0.2 | 1.05 |
| rs10759243 | 9 | C/A | 0.99 (0.82, 1.20) 0.9 | 1.05 |
| rs865686 | 9 | T/G | 0.97 (0.81, 1.16) 0.8 | 1.11 |
| rs2380205 | 10 | C/T | 1.00 (0.84, 1.19) 1.0 | 1.02 |
| rs7072776 | 10 | G/A | 1.03 (0.85, 1.24) 0.8 | 1.06 |
| rs11814448 | 10 | A/C | 0.88 (0.45, 1.73) 0.7 | 1.21 |
| rs10995190 | 10 | G/A | 0.91 (0.71, 1.15) 0.4 | 1.17 |
| rs704010 | 10 | C/T | 0.86 (0.72, 1.03) 0.09 | 1.07 |
| rs7904519 | 10 | A/G | 0.99 (0.83, 1.17) 0.8 | 1.06 |
| rs11199914 | 10 | C/T | 0.90 (0.75, 1.09) 0.3 | 1.06 |
| rs2981579 | 10 | G/A | 1.05 (0.88, 1.24) 0.6 | 1.25 |
| rs3817198 | 11 | T/C | 1.17 (0.97, 1.40) 0.1 | 1.07 |
| rs3903072 | 11 | G/T | 0.92 (0.77, 1.09) 0.3 | 1.06 |
| rs78540526 | 11 | C/T | 1.20 (0.88, 1.64) 0.3 | 1.18 |
| rs554219 | 11 | C/G | 1.12 (0.88, 1.44) 0.4 | 1.12 |
| rs75915166 | 11 | C/A | 0.99 (0.70, 1.38) 0.9 | 1.02 |
| rs11820646 | 11 | C/T | 0.89 (0.74, 1.06) 0.2 | 1.05 |
| rs12422552 | 12 | G/C | 1.00 (0.82, 1.21) 1.0 | 1.03 |
| rs10771399 | 12 | A/G | 0.69 (0.53, 0.89) 0.005 | 1.16 |
| rs17356907 | 12 | A/G | 0.85 (0.70, 1.03) 0.1 | 1.10 |
| rs1292011 | 12 | A/G | 1.00 (0.84, 1.20) 1.0 | 1.08 |
| rs11571833 | 13 | A/T | 1.34 (0.61, 2.94) 0.5 | 1.26 |
| rs2236007 | 14 | G/A | 0.92 (0.75, 1.14) 0.5 | 1.09 |
| rs2588809 | 14 | C/T | 1.01 (0.81, 1.26) 0.9 | 1.07 |
| rs999737 | 14 | C/T | 1.11 (0.90, 1.37) 0.3 | 1.08 |
| rs941764 | 14 | A/G | 1.03 (0.86, 1.24) 0.7 | 1.06 |
| rs3803662 | 16 | G/A | 1.27 (1.05, 1.55) 0.02 | 1.23 |
| rs17817449 | 16 | T/G | 0.94 (0.79, 1.13) 0.5 | 1.08 |
| rs11075995 | 16 | A/T | 1.12 (0.91, 1.38) 0.3 | 1.04 |
| rs13329835 | 16 | A/G | 1.02 (0.83, 1.25) 0.9 | 1.08 |
| rs6504950 | 17 | G/A | 0.93 (0.78, 1.12) 0.4 | 1.07 |
| rs527616 | 18 | G/C | 0.85 (0.71, 1.01) 0.07 | 1.04 |
| rs1436904 | 18 | T/G | 1.05 (0.88, 1.25) 0.6 | 1.06 |
| rs8170 | 19 | G/A | 1.14 (0.92, 1.42) 0.2 | 1.03 |
| rs2363956 | 19 | G/T | 0.98 (0.83, 1.16) 0.8 | 1.03 |
| rs4808801 | 19 | A/G | 0.92 (0.77, 1.10) 0.3 | 1.07 |
| rs3760982 | 19 | G/A | 1.00 (0.84, 1.19) 1.0 | 1.06 |
| rs2823093 | 21 | G/A | 1.02 (0.84, 1.25) 0.8 | 1.08 |
| rs17879961 | 22 | A/G | – | 1.36 |
| rs132390 | 22 | T/C | 0.88 (0.53, 1.44) 0.6 | 1.11 |
| rs6001930 | 22 | T/C | 1.43 (1.07, 1.91) 0.02 | 1.13 |

^*^ Major/minor allele; ^†^ per allele, adjusted for age group; ‡ Published ORs from Mavaddat N, Pharoah PD, Michailidou K, Tyrer J, Brook MN, Bolla MK, et al. Prediction of breast cancer risk based on profiling with common genetic variants. J Natl Cancer Inst. 2015;107:djv036.
