# Supplementary material for: A streamlined model for use in clinical breast cancer risk assessment maintains predictive power and is further improved with inclusion of a polygenic risk score
Source: PLoS One. 2021 Jan 22;16(1):e0245375. doi: 10.1371/journal.pone.0245375 (PMC7822550; doi:10.1371/journal.pone.0245375)
Supplement: S4 Table — (DOCX) [file pone.0245375.s004.docx]

S4 Table. Unadjusted ORs for individual SNPs in African Americans

| **SNP** | **Chromosome** | **Alleles^*^** | **OR^†^ (95% CI) *P*** | **Published OR^a^** | **(95% CI)** |
| --- | --- | --- | --- | --- | --- |
| rs11249433 | 1 | G/A | 1.19 (0.94, 1.49) 0.1 | 0.99 | (0.88-1.12) |
| rs4245739 | 1 | C/A | 1.01 (0.86, 1.18) 1.0 | 0.97 | (0.88-1.06) |
| rs616488 | 1 | A/G | 1.03 (0.83, 1.27) 0.8 | 1.03 | (0.92-1.15) |
| rs6678914 | 1 | G/A | 1.05 (0.90, 1.22) 0.5 | 1.00 | (0.92-1.08) |
| rs1045485 | 2 | C/G | 1.22 (0.94, 1.59) 0.1 | 0.99 | (0.84-1.15) |
| rs12710696 | 2 | A/G | 1.11 (0.96, 1.27) 0.2 | 1.06 | (0.98-1.14) |
| rs13387042 | 2 | A/G | 1.11 (0.95, 1.30) 0.2 | 1.12 | (1.03-1.23) |
| rs1550623 | 2 | A/G | 1.11 (0.95, 1.30) 0.2 | 1.10 | (1.01-1.20) |
| rs16857609 | 2 | A/G | 1.15 (0.98, 1.34) 0.09 | 1.17 | (1.07-1.28) |
| rs2016394 | 2 | G/A | 1.02 (0.85, 1.23) 0.8 | 1.05 | (0.96-1.14) |
| rs4849887 | 2 | G/A | 1.27 (1.08, 1.49) 0.004 | 1.16 | (1.06-1.26) |
| rs12493607 | 3 | G/C | 1.07 (0.88, 1.33) 0.5 | 1.04 | (0.93-1.17) |
| rs4973768 | 3 | A/G | 1.05 (0.90, 1.21) 0.6 | 1.04 | (0.96-1.12) |
| rs6762644 | 3 | G/A | 1.05 (0.91, 1.20) 0.5 | 1.05 | (0.97-1.13) |
| rs6828523 | 4 | C/A | 1.07 (0.92, 1.23) 0.4 | 1.00 | (0.92-1.08) |
| rs9790517 | 4 | A/G | 0.86 (0.65, 1.14) 0.3 | 0.88 | (0.76-1.02) |
| rs10069690 | 5 | A/G | 1.22 (0.97, 1.53) 0.09 | 1.13 | (1.04-1.22) |
| rs10472076 | 5 | G/A | 1.13 (0.97, 1.32) 0.1 | 0.95 | (0.87-1.04) |
| rs10941679 | 5 | G/A | 1.22 (1.03, 1.45) 0.02 | 1.04 | (0.95-1.15) |
| rs1353747 | 5 | A/C | 0.98 (0.61, 1.56) 0.9 | 1.01 | (0.77-1.33) |
| rs1432679 | 5 | G/A | 1.11 (0.93, 1.33) 0.2 | 1.07 | (0.97-1.18) |
| rs4415084 | 5 | T/C | 1.04 (0.90, 1.20) 0.6 | 1.10^b^ | (0.98-1.22) |
| rs889312 | 5 | C/A | 1.14 (0.99, 1.32) 0.07 | 1.07 | (0.98-1.16) |
| rs11242675 | 6 | A/G | 0.83 (0.72, 0.96) 0.009 | 1.06 | (0.98-1.15) |
| rs17529111 | 6 | G/A | 1.12 (0.85, 1.47) 0.4 | 0.99 | (0.85-1.15 |
| rs204247 | 6 | G/A | 0.97 (0.84, 1.13) 0.7 | 1.13 | (1.04-1.22) |
| rs2046210 | 6 | A/G | 1.07 (0.93, 1.24) 0.3 | 0.99 | (0.92-1.07) |
| rs3757318 | 6 | A/G | 1.00 (0.67, 1.49) 1.0 | 1.11 | (0.91-1.36) |
| rs720475 | 7 | G/A | 0.98 (0.79, 1.22) 0.9 | 0.99 | (0.88-1.11) |
| rs11780156 | 8 | A/G | 1.33 (0.98, 1.79) 0.06 | 0.84 | (0.70-1.01) |
| rs13281615 | 8 | G/A | 1.14 (0.98, 1.31) 0.08 | 1.06 | (0.98-1.14) |
| rs2943559 | 8 | G/A | 0.85 (0.72, 1.01) 0.06 | 1.07 | (0.98-1.18) |
| rs6472903 | 8 | A/C | 1.21 (0.93, 1.57) 0.2 | 1.02 | (0.90-1.16) |
| rs9693444 | 8 | A/C | 1.17 (1.01, 1.35) 0.03 | 1.06 | (0.98-1.15) |
| rs1011970 | 9 | A/C | 1.19 (1.02, 1.38) 0.02 | 1.06 | (0.98-1.15) |
| rs10759243 | 9 | A/C | 0.97 (0.84, 1.12) 0.7 | 1.02 | (0.94-1.10) |
| rs865686 | 9 | A/C | 1.11 (0.96, 1.28) 0.2 | 1.09 | (1.01-1.17) |
| rs10995190 | 10 | G/A | 0.88 (0.73, 1.05) 0.2 | 0.98 | (0.88-1.08) |
| rs11199914 | 10 | G/A | 0.90 (0.78, 1.03) 0.1 | 0.97 | (0.90-1.05) |
| rs11814448 | 10 | C/A | 1.05 (0.91, 1.21) 0.5 | 1.04 | (0.96-1.08) |
| rs2380205 | 10 | G/A | 1.09 (0.95, 1.26) 0.2 | 0.98 | (0.90-1.06) |
| rs2981579 | 10 | A/G | 1.13 (0.97, 1.33) 0.2 | 1.18 | (1.09-1.27) |
| rs2981582 | 10 | A/G | 1.00 (0.87, 1.15) 1.0 | 1.05^b^ | (0.92-1.19) |
| rs704010 | 10 | A/G | 0.95 (0.75, 1.20) 0.7 | 0.98 | (0.87-1.12) |
| rs7072776 | 10 | A/G | 0.91 (0.79, 1.05) 0.2 | 1.04 | (0.97-1.13) |
| rs7904519 | 10 | G/A | 1.00 (0.85, 1.19) 1.0 | 1.13 | (1.03-1.25) |
| rs11820646 | 11 | G/A | 1.05 (0.89, 1.24) 0.5 | 0.98 | (0.89-1.07) |
| rs3817198 | 11 | G/A | 1.14 (0.95, 1.36) 0.2 | 0.98 | (0.89-1.09) |
| rs3903072 | 11 | C/A | 0.96 (0.80, 1.15) 0.7 | 0.99 | (0.89-1.10) |
| rs554219 | 11 | G/C | 1.07 (0.90, 1.28) 0.4 | 1.00 | (0.91-1.10) |
| rs614367 | 11 | A/G | 1.19 (0.98, 1.45) 0.08 | 0.96 | (0.86-1.08) |
| rs75915166 | 11 | A/C | 2.01 (1.22, 3.32) 0.006 | 1.44 | (1.04-1.97) |
| rs10771399 | 12 | A/G | 1.11 (0.77, 1.62) 0.6 | 1.19 | (0.97-1.46) |
| rs12422552 | 12 | C/G | 0.99 (0.85, 1.15) 0.9 | 1.02 | (0.94-1.10) |
| rs1292011 | 12 | A/G | 1.03 (0.89, 1.19) 0.7 | 1.03 | (0.95-1.11) |
| rs17356907 | 12 | A/G | 1.17 (0.98, 1.40) 0.09 | 1.02 | (0.93-1.12) |
| rs11571833 | 13 | A/T | 1.23 (0.48, 3.11) 0.7 | 0.95 | (0.44-2.03) |
| rs2236007 | 14 | G/A | 0.72 (0.57, 0.92) 0.007 | 0.90 | (0.78-1.04) |
| rs2588809 | 14 | A/G | 0.99 (0.85, 1.16) 0.9 | 1.01 | (0.93-1.10) |
| rs941764 | 14 | G/A | 0.94 (0.81, 1.10) 0.5 | 1.10 | (1.01-1.20) |
| rs999737 | 14 | G/A | 1.06 (0.78, 1.44) 0.7 | 1.03 | (0.85-1.24) |
| rs11075995 | 16 | A/T | 1.01 (0.83, 1.22) 1.0 | 1.07 | (0.97-1.18) |
| rs13329835 | 16 | G/A | 0.98 (0.85, 1.13) 0.8 | 1.08 | (0.99-1.17) |
| rs17817449 | 16 | A/C | 1.11 (0.96, 1.28) 0.2 | 1.05 | (0.97-1.13) |
| rs3803662 | 16 | A/G | 1.05 (0.91, 1.21) 0.5 | 0.99 | (0.92-1.07) |
| rs6504950 | 17 | G/A | 1.11 (0.96, 1.29) 0.2 | 1.06 | (0.98-1.15) |
| rs1436904 | 18 | A/C | 1.09 (0.93, 1.29) 0.3 | 0.98 | (0.90-1.07) |
| rs527616 | 18 | C/G | 0.92 (0.74, 1.13) 0.4 | 0.98 | (0.87-1.10) |
| rs3760982 | 19 | A/G | 1.04 (0.90, 1.19) 0.6 | 1.00 | (0.93-1.08) |
| rs4808801 | 19 | A/G | 1.05 (0.91, 1.22) 0.5 | 1.01 | (0.93-1.10) |
| rs8170 | 19 | A/G | 1.05 (0.87, 1.26) 0.5 | 1.13 | (1.02-1.24) |
| rs2284378 | 20 | T/C | 0.92 (0.76, 1.12) 0.4 | 1.06 | (0.95-1.17) |
| rs2823093 | 21 | G/A | 1.00 (0.86, 1.16) 1.0 | 1.03 | (0.95-1.11) |
| rs132390 | 22 | G/A | 0.90 (0.58, 1.42) 0.7 | 0.88 | (0.72-1.07) |
| rs6001930 | 22 | G/A | 1.06 (0.87, 1.30) 0.6 | 1.02 | (0.91-1.14) |

^*^ risk allele/reference allele; ^†^ per allele

^a^ Feng et al (15)

^b^ Palmer et al (17)
