# Supplementary material for: A streamlined model for use in clinical breast cancer risk assessment maintains predictive power and is further improved with inclusion of a polygenic risk score
Source: PLoS One. 2021 Jan 22;16(1):e0245375. doi: 10.1371/journal.pone.0245375 (PMC7822550; doi:10.1371/journal.pone.0245375)
Supplement: S5 Table — (DOCX) [file pone.0245375.s005.docx]

S5 Table. Unadjusted ORs for individual SNPs in Hispanics

| **SNP** | **Chromosome** | **Alleles^*^** | **OR^†^ (95% CI) *P*** | **Published OR** | **(95% CI)** |
| --- | --- | --- | --- | --- | --- |
| rs11249433 | 1 | C/T | 1.18 (0.84, 1.66) 0.3 | 1.10 | (1.08-1.12) |
| rs11552449 | 1 | T/C | 0.89 (0.68, 1.17) 0.4 | 1.08 | (1.05-1.11) |
| rs4245739 | 1 | A/C | 0.98 (0.76, 1.27) 0.9 | 1.13 | (1.09-1.18) |
| rs616488 | 1 | G/A | 0.83 (0.65, 1.06) 0.1 | 0.94 | (0.92-0.96) |
| rs6678914 | 1 | A/G | 0.99 (0.77, 1.28) 1.0 | 1.08 | (1.04-1.12) |
| rs12710696 | 2 | T/C | 0.97 (0.75, 1.24) 0.8 | 1.10 | (1.06-1.15) |
| rs13387042 | 2 | A/G | 1.05 (0.83, 1.33) 0.7 | 0.88 | (0.86-0.90) |
| rs1550623 | 2 | G/A | 0.97 (0.72, 1.29) 0.8 | 0.94 | (0.92-0.97) |
| rs16857609 | 2 | T/C | 1.02 (0.80, 1.30) 0.9 | 1.07 | (1.05-1.10) |
| rs2016394 | 2 | A/G | 0.95 (0.72, 1.24) 0.7 | 0.95 | (0.93-0.97) |
| rs4849887 | 2 | T/C | 0.90 (0.62, 1.30) 0.6 | 0.91 | (0.88-0.94) |
| rs12493607 | 3 | G/G | 1.07 (0.83, 1.36) 0.6 | 1.05 | (1.03-1.08) |
| rs4973768 | 3 | T/C | 0.95 (0.75, 1.20) 0.6 | 1.09 | (1.07-1.12) |
| rs6762644 | 3 | C/A | 1.00 (0.77, 1.30) 1.0 | 1.07 | (1.04-1.09) |
| rs6828523 | 4 | A/C | 0.87 (0.64, 1.18) 0.4 | 0.91 | (0.88-0.94) |
| rs7696175 | 4 | T/C | 1.02 (0.80, 1.31) 0.9 | 0.89^a^ | (0.78-1.00) |
| rs9790517 | 4 | T/C | 1.06 (0.82, 1.38) 0.6 | 1.05 | (1.03-1.08) |
| rs10069690 | 5 | T/C | 1.19 (0.73, 1.95) 0.5 | 1.18 | (1.13-1.25) |
| rs10472076 | 5 | C/T | 1.12 (0.86, 1.45) 0.4 | 1.05 | (1.03-1.07) |
| rs10941679 | 5 | G/A | 1.42 (1.09, 1.85) 0.008 | 1.12 | (1.09-1.15) |
| rs1353747 | 5 | G/T | 0.85 (0.56, 1.30) 0.5 | 0.92 | (0.89-0.96) |
| rs1432679 | 5 | C/T | 1.28 (1.01, 1.63) 0.04 | 1.07 | (1.04-1.09) |
| rs2067980 | 5 | G/A | 1.38 (0.96, 1.97) 0.08^b^ | 1.00 | (0.87-1.13) |
| rs889312 | 5 | C/A | 1.14 (0.89, 1.45) 0.3 | 1.12 | (1.09-1.14) |
| rs11242675 | 6 | C/T | 1.03 (0.81, 1.30) 0.8 | 0.94 | (0.92-0.96) |
| rs140068132 | 6 | G/A | 0.73 (0.42, 1.28) 0.3 | 0.63^a^ | (0.53-0.75) |
| rs204247 | 6 | G/A | 1.05 (0.83, 1.33) 0.7 | 1.05 | (1.03-1.07) |
| rs2046210 | 6 | A/G | 1.10 (0.86, 1.41) 0.5 | 1.05 | (1.02-1.07) |
| rs2180341 | 6 | G/A | 1.01 (0.76, 1.32) 1.0 | 1.00^a^ | (0.89-1.12) |
| rs17157903 | 7 | T/C | 0.94 (0.65, 1.36) 0.7 | 0.93^b^ | (0.80-1.09) |
| rs720475 | 7 | A/G | 0.84 (0.61, 1.15) 0.3 | 0.94 | (0.92-0.96) |
| rs11780156 | 8 | T/C | 0.91 (0.66, 1.25) 0.6 | 1.07 | (1.04-1.10) |
| rs13281615 | 8 | G/A | 1.18 (0.93, 1.49) 0.2 | 1.10 | (1.07-1.12) |
| rs2943559 | 8 | G/A | 1.09 (0.73, 1.64) 0.7 | 1.13 | (1.09-1.18) |
| rs6472903 | 8 | G/T | 0.72 (0.45, 1.15) 0.2 | 0.91 | (0.89-0.94) |
| rs9693444 | 8 | A/C | 0.92 (0.72, 1.18) 0.5 | 1.07 | (1.05-1.10) |
| rs1011970 | 9 | T/G | 1.03 (0.81, 1.32) 0.8 | 1.05 | (1.02-13.08) |
| rs10759243 | 9 | A/C | 1.02 (0.81, 1.30) 0.8 | 1.06 | (1.03-1.08) |
| rs865686 | 9 | T/G | 1.30 (0.99, 1.65) 0.06 | 0.90 | (0.88-0.92) |
| rs10995190 | 10 | G/A | 0.95 (0.66, 1.37) 0.8 | 0.86 | (0.83-0.88) |
| rs11199914 | 10 | T/C | 1.84 (0.66, 1.07) 0.2 | 0.95 | (0.93-0.97 |
| rs11814448 | 10 | C/A | 0.86 (0.55, 1.34) 0.5 | 1.22 | (1.17-1.35) |
| rs2380205 | 10 | C/T | 0.91 (0.72, 1.15) 0.4 | 0.98 | (0.96-1.00) |
| rs2981579 | 10 | T/C | 0.88 (0.69, 1.13) 0.3 | 1.25 | (1.22-1.28) |
| rs2981582 | 10 | T/C | 0.93 (0.73, 1.19) 0.6 | 1.19^b^ | (1.05-1.36) |
| rs704010 | 10 | T/C | 1.16 (0.92, 1.47) 0.2 | 1.07 | (1.05-1.09) |
| rs7072776 | 10 | A/G | 0.82 (0.64, 1.06) 0.1 | 1.06 | (1.03-1.08) |
| rs11820646 | 11 | T/C | 0.93 (1.73, 1.17) 0.5 | 0.95 | (0.93-0.97) |
| rs3817198 | 11 | C/T | 1.24 (0.95, 1.62) 0.1 | 1.07 | (1.05-1.10) |
| rs3903072 | 11 | T/G | 1.07 (0.84, 1.36) 0.6 | 0.94 | (0.92-0.96) |
| rs10771399 | 12 | T/C | 1.41 (0.88, 2.27) 0.2 | 0.86 | (0.83-0.89) |
| rs12422552 | 12 | C/G | 1.18 (0.89, 1.56) 0.2 | 1.05 | (1.03-1.07) |
| rs1292011 | 12 | A/G | 1.00 (0.79, 1.28) 1.0 | 0.92 | (0.90-0.94) |
| rs17356907 | 12 | G/A | 0.79 (0.61, 1.02) 0.07 | 0.91 | (0.89-0.93) |
| rs2236007 | 14 | A/G | 0.63 (0.43, 0.94) 0.02 | 0.92 | (0.90-0.94) |
| rs2588809 | 14 | T/C | 0.98 (0.73, 1.33) 0.9 | 1.08 | (1.05-1.11) |
| rs941764 | 14 | G/A | 0.90 (0.71, 1.14) 0.4 | 1.06 | (1.04-1.09) |
| rs999737 | 14 | C/T | 1.05 (0.77, 1.44) 0.8 | 0.92 | (0.90-0.94) |
| rs11075995 | 16 | A/T | 1.34 (1.05, 1.71) 0.02 | 1.10 | (1.05-1.15) |
| rs13329835 | 16 | G/A | 1.31 (1.01, 1.70) 0.04 | 1.08 | (1.05-1.10) |
| rs17817449 | 16 | G/T | 1.07 (0.83, 1.37) 0.6 | 0.93 | (0.91-0.95) |
| rs3803662 | 16 | T/C | 1.14 (0.90, 1.45) 0.3 | 1.23 | (1.20-1.26) |
| rs6504950 | 17 | G/A | 1.06 (0.80, 1.41) 0.7 | 0.93 | (0.91-0.96) |
| rs1436904 | 18 | G/T | 0.93 (0.73, 1.18) 0.5 | 0.96 | (0.94-0.98) |
| rs527616 | 18 | C/G | 1.01 (0.76, 1.33) 1.0 | 0.95 | (0.93-0.97) |
| rs2363956 | 19 | C/A | 1.05 (0.82, 1.34) 0.7 | 1.03 | (1.00-1.05) |
| rs3760982 | 19 | A/G | 0.98 (0.77, 1.25) 0.9 | 1.06 | (1.04-1.08) |
| rs4808801 | 19 | G/A | 0.84 (0.66, 1.08) 0.2 | 0.93 | (0.91-0.96) |
| rs8170 | 19 | A/G | 0.64 (0.42, 0.97) 0.04 | 1.03 | (1.00-1.05) |
| rs2823093 | 21 | G/A | 1.27 (0.96, 1.67) 1.0 | 0.93 | (0.91-0.95) |
| rs6001930 | 22 | C/T | 1.31 (0.90, 1.90) 0.2 | 1.13 | (1.10-1.17) |

^*^ tested allele/reference allele; ^†^ per allele

^a^ Fejerman et al (24)

^b^ Fejerman et al (18)
